# Differential Gene Expression in Host Ubiquitination Processes in Childhood Malarial Anemia

**DOI:** 10.3389/fgene.2021.764759

**Published:** 2021-11-22

**Authors:** Samuel B. Anyona, Evans Raballah, Qiuying Cheng, Ivy Hurwitz, Caroline Ndege, Elly Munde, Walter Otieno, Philip D. Seidenberg, Kristan A. Schneider, Christophe G. Lambert, Benjamin H. McMahon, Collins Ouma, Douglas J. Perkins

**Affiliations:** ^1^ Department of Medical Biochemistry, School of Medicine, Maseno University, Maseno, Kenya; ^2^ University of New Mexico-Kenya Global Health Programs, Kisumu and Siaya, Kenya; ^3^ Department of Medical Laboratory Sciences, School of Public Health Biomedical Sciences and Technology, Masinde Muliro University of Science and Technology, Kakamega, Kenya; ^4^ Center for Global Health, University of New Mexico, Albuquerque, NM, United States; ^5^ Department of Clinical Medicine, School of Health Science, Kirinyaga University, Kerugoya, Kenya; ^6^ Department of Pediatrics and Child Health, School of Medicine, Maseno University, Maseno, Kenya; ^7^ Department of Emergency Medicine, University of New Mexico, Albuquerque, NM, United States; ^8^ Department Applied Computer and Bio-Sciences, University of Applied Sciences Mittweida, Mittweida, Germany; ^9^ Theoretical Biology and Biophysics Group, Theoretical Division, Los Alamos National Laboratory, Los Alamos, NM, United States; ^10^ Department of Biomedical Sciences and Technology, School of Public Health and Community Development, Maseno University, Maseno, Kenya

**Keywords:** ubiquitination, ubiquitin proteasome system, differential gene expression, *Plasmodium falciparum*, malarial anemia

## Abstract

**Background:** Malaria remains one of the leading global causes of childhood morbidity and mortality. In holoendemic *Plasmodium falciparum* transmission regions, such as western Kenya, severe malarial anemia [SMA, hemoglobin (Hb) < 6.0 g/dl] is the primary form of severe disease. Ubiquitination is essential for regulating intracellular processes involved in innate and adaptive immunity. Although dysregulation in ubiquitin molecular processes is central to the pathogenesis of multiple human diseases, the expression patterns of ubiquitination genes in SMA remain unexplored.

**Methods:** To examine the role of the ubiquitination processes in pathogenesis of SMA, differential gene expression profiles were determined in Kenyan children (*n* = 44, aged <48 mos) with either mild malarial anemia (M*l*MA; Hb ≥9.0 g/dl; *n* = 23) or SMA (Hb <6.0 g/dl; *n* = 21) using the Qiagen Human Ubiquitination Pathway RT^2^ Profiler PCR Array containing a set of 84 human ubiquitination genes.

**Results:** In children with SMA, 10 genes were down-regulated (*BRCC3*, *FBXO3*, *MARCH5*, *RFWD2*, *SMURF2*, *UBA6*, *UBE2A*, *UBE2D1*, *UBE2L3*, *UBR1*), and five genes were up-regulated (*MDM2*, *PARK2*, *STUB1*, *UBE2E3*, *UBE2M*). Enrichment analyses revealed Ubiquitin-Proteasomal Proteolysis as the top disrupted process, along with altered sub-networks involved in proteasomal, protein, and ubiquitin-dependent catabolic processes.

**Conclusion:** Collectively, these novel results show that protein coding genes of the ubiquitination processes are involved in the pathogenesis of SMA.

## Introduction

An estimated number of malaria cases reported worldwide in 2019 were 229 million, with the African region accounting for 215 million (94%) of all malaria incidents (WHO, 2020). Globally, malaria caused an estimated 409,000 mortalities, with Africa recording 384,000 of the total deaths, 99.7% of which were attributable to *Plasmodium falciparum* infections (WHO, 2020). Sixty seven percent of the mortalities were reported in children under the age of 5 years, and 94% occurred in the WHO African region (WHO, 2020). In Kenya, 34.4 million (75%) of the population is at risk of malaria. In 2019, an estimated 4.7 million confirmed malaria cases were reported, resulting in 12,652 deaths (WHO, 2020), with a constant mortality rate in children under-five years of age remaining at 52 deaths for every 1,000 live births (Kenya Malaria Impact Eval, 2017). In holoendemic *P. falciparum* transmission areas such as western Kenya, malaria infections continue to be among the principal causes of childhood morbidity and mortality (Amek et al., 2014; Amboko et al., 2016; Amek et al., 2018). In this region, children present with severe malarial anemia [SMA, hemoglobin (Hb) < 5.0 g/dl] as the primary severe disease manifestation of *P. falciparum* infections (Zucker et al., 1997; Breman et al., 2001; Obonyo et al., 2007), often compounded by features of respiratory distress and hypoglycemia, while cerebral malaria is rarely reported (Breman et al., 2001; Rowe et al., 2009; Okiro et al., 2010). In addition, co-infections, nutritional deficiencies, and host and/or parasite genetic factors influence the severity of malarial infections (Kendall et al., 1980; Aluoch, 1997; Aidoo et al., 2002; Otieno et al., 2006; Kifude et al., 2007; Davenport et al., 2010; Waitumbi et al., 2010; Were et al., 2011). The pathogenesis of SMA is multifaceted, and can be ascribed, at least in part, to altered host immune responses which suppress erythroid development and enhance hemolysis, resulting in markedly low hemoglobin concentrations (Fendel et al., 2010; Perkins et al., 2011; Ong’echa et al., 2011; Davenport et al., 2012).

To date, no studies have reported the role of the human ubiquitin proteasome system (UPS) on the pathogenesis of malaria. The UPS is the main cellular machinery responsible for the degradation of intracellular proteins in eukaryotic cells, and plays a central role in the regulation of cellular processes, including proliferation, cell-cycle control, transcriptional regulation, and stress-responses [reviewed in (Glickman and Ciechanover, 2002)]. In most mammalian cells, the UPS degrades more than 90% of proteins, ensuring that misfolded, oxidized, or damaged proteins, which possess intrinsic toxicity, are degraded (Schubert et al., 2000; Bucciantini et al., 2002; Pagan et al., 2013).

Since appropriate transcriptional regulation of the UPS is important for the maintenance of cellular homeostasis, perturbations in the expression of UPS genes can cause cellular cataclysm and pathology (Coux et al., 1996; Hilt and Wolf, 1996; Ciechanover, 1998; Voges et al., 1999; Tanaka, 2013). For cells to overcome transcriptional challenges, the proteolytic and non-proteolytic activities of the UPS are biologically conserved features. Transcriptional activators and coactivators are the major intervention point of the UPS, which regulate ubiquitin/proteasome dependent processing, and modulate targeted gene expression (Hoppe et al., 2000; Muratani and Tansey, 2003; Geng et al., 2012). In addition, the UPS is important for antigen processing during host-pathogen interactions, which contribute to changes in both ubiquitin and proteasomes that are associated with various clinical syndromes (Llovera et al., 1998; Edelmann and Kessler, 2008; Sixt and Dahlmann, 2008; Seissler et al., 2017).

A large network of proteins involved in ubiquitination and ubiquitin-mediated degradation by the 26S proteasome constitutes the UPS (Ciechanover, 2005; Finley and Prado, 2020). Through the UPS, the proteasome regulates all major aspects of cellular processes, such as the cell cycle, gene expression, signal transduction, immune response, apoptosis and carcinogenesis (Ciechanover, 2005). To serve as the single terminal “modulator” for numerous ubiquitination pathways, the proteasome evolved remarkable flexibility to vigorously organize receptors of the ubiquitin molecule, transport factors, multiple enzyme systems such as the ATPases, deubiquitinases, and ubiquitin ligases, as well as a large network of proteins to permit recognition of the substrates, efficient processing, and accuracy of intracellular regulation of the target protein (Finley and Prado, 2020).

Ubiquitination is a post-translational modification of the lysine residue at the ε-amino group by the covalent attachment of a single or multiple ubiquitin monomers (Geng et al., 2012). During the ubiquitination process, multiple ubiquitin proteins can be covalently bound to target proteins by the ubiquitination enzyme cascade, which include the ubiquitin activating enzyme (E1), ubiquitin carrier protein (E2), and ubiquitin protein ligase (E3) (Ciechanover, 1998). Theses enzymes transfer ubiquitin molecules to cellular targets in a sequential manner (Pickart, 2001). Specifically, E1 activates ubiquitin in an ATP-hydrolyzing reaction in which the C-terminus of ubiquitin forms a thioester bond with the catalytic cysteine of an E1 ubiquitin activating enzyme (Pickart and Eddins, 2004). Ubiquitin is then transported from E1 to the catalytic cysteine residue of the E2 active site. The E2-ubiquitin conjugate then cooperates with E3 to transfer ubiquitin to the substrate (Pickart and Eddins, 2004). The E3s confer specificity to ubiquitination by recognizing target substrates, with the activity of most E3s being specified by domains of really interesting new gene (*RING*) (Pickart and Eddins, 2004). As such, the RING domain E3s mediate the interaction of the E2-ubiquitin complex to facilitate the transfer of the C-terminus of ubiquitin to a substrate lysine to form an isopeptide bond (Pickart and Eddins, 2004). In addition, the E3s act through E6-AP carboxyl terminus (*HECT*) domains. In the HECT E3-dependent reactions, ubiquitin from the E2-ubiquitin conjugate is transferred onto a catalytic E3 cysteine, which then transfers the C-terminus of ubiquitin to a substrate lysine, forming an isopeptide bond (Pickart and Eddins, 2004). These processes may attach either a single ubiquitin molecule on one acceptor site (mono-ubiquitination) or multiple ubiquitin molecules (polyubiquitination) (Sadowski et al., 2012).

Since ubiquitination is essential for regulating physiological processes, including innate and adaptive immunity, and alterations in ubiquitin molecular pathways are central to the pathogenesis of several human diseases [reviewed in (Popovic et al., 2014)], we investigated the impact of ubiquitination process on disease severity in children with *P. falciparum* malaria. As an initial step, we determined the transcriptional changes of 84 (key) human ubiquitination genes in Kenyan children (*n* = 44) with mild malaria anemia (M*l*MA; Hb ≥9.0 g/dl; *n* = 23) and severe malarial anemia (SMA; Hb <6.0 g/dl; *n* = 21). Results presented here show that children with SMA have dysregulation in genes that regulate human ubiquitination processes, thereby identifying previously undiscovered molecular networks involved in the pathogenesis of malarial anemia.

## Methods

### Study Area

The study was conducted at Siaya County Referral Hospital (SCRH) catchment area, a holoendemic *P. falciparum* transmission region located in western Kenya. Details of the region have previously been published (Ong’echa et al., 2006). One of the primary causes of childhood mortality and morbidity in the Siaya community is *P. falciparum malaria* (Perkins et al., 2013). Individuals inhabiting the study area are predominantly from the Luo ethnic group (>96%), a culturally and biologically homogeneous population (Bloland et al., 1999).

### Study Design and Participants

Children (<48 mos) were enrolled in a short-term prospective observational study between (Mar 2017-Aug 2018) to investigate host-genetic factors associated with community-based acquisition of pediatric infectious diseases. Inclusion criteria for enrollment included: presence of axillary temperature ≥37.5°C, parent/legal guardian providing informed written consent and willingness to attend day 14 (well visit) appointment and living within 25 km from the hospital. Children hospitalized for suspected non-infectious causes such as injury and/or accident were excluded. All study participants were scheduled for a follow-up well visit on day 14 after enrollment to assess their health status. Children who failed to return for scheduled visits were located by our community health team to assess the child’s health status. Parents/legal guardians were asked to return their child to hospital in-case of any acute illnesses prior to their day-14 well visit. Patients were managed according to the Ministry of Health-Kenya guidelines. Demographic, clinical and laboratory measures for each study participant were collected upon enrollment, during hospitalization, and at the 14 days follow-up visit.

### Laboratory Investigations

Following consent and enrollment, venous blood samples (≤3.0 ml) were collected in EDTA-containing vacutainer tubes, prior to treatment. Complete blood counts (CBC) were determined using a Beckman Coulter AcT diff2 (Beckman-Coulter Corporation, Miami, FL, United States). Malaria parasite densities were determined by microscopy according to our published methods (Ong’echa et al., 2006). Children with *P. falciparum* infections (any density parasitemia) were stratified based on hemoglobin concentrations to represent distinct and non-overlapping groups: Hb ≥9.0 g/dl (mild malarial anemia, M*l*MA, *n* = 23) and Hb <6.0 g/dl (severe malarial anemia, SMA, *n* = 21). Defining SMA as Hb <6.0 g/dl, rather than Hb <5.0 g/dl, was based on a previous longitudinal study that investigated the distribution of anemia using >14,000 Hb measurements in age- and gender-matched children from the same geographical location (McElroy et al., 1999). This definition also provided the most appropriate distribution of Hb concentrations in the cohort and offered the ability to have a balanced sample size for robust conclusions from the differential expression analyses. To delineate other factors that can contribute to the development of anemia in children residing in the region, HIV-1, bacteremia, and sickle-cell trait status were determined per our published methods (Otieno et al., 2006; Were et al., 2011). Whole blood samples (500 µl) from each study participant were preserved in an equal volume of Trizol^®^ Reagent (Thermo Fisher Scientific, Waltham, MA, United States) and stored at −80°C until use.

### Isolation of RNA From Patient Samples

Total RNA was isolated from Trizol^®^ Reagent preserved patient whole blood (500 µl) using E. Z.N.A.^®^ PX Blood RNA Kit (Omega Bio-Tek Inc., Norcross, GA, United States), and treated with RNase-free DNase I (New England Biolabs, Ipswich, MA, United States) to remove any contaminating DNA following the manufacturer’s instructions.

Total RNA was further cleaned using the RNA Clean & Concentrator Kit (ZYMO Research Corp., United States). The quantity of RNA was measured using NanoDrop 2000 Spectrophotometer (Thermo Fisher Scientific, Waltham, MA, United States), while the quality and integrity of the purified RNA was estimated using an automated Agilent 2100 Bioanalyzer (Agilent Technologies, Santa Clara, CA, United States). Samples with RNA integrity number (RIN) ≥8 were used for cDNA synthesis.

### Synthesis of Complementary DNAs

The cDNAs were synthesized using the RT^2^ First Stand Kit (Qiagen, LLC-USA, Germantown, MD, United States) in a 2-step procedure that involved genomic DNA elimination and reverse transcription according to the manufacturers’ protocol. The RT^2^ First Strand Kits used for the data presented were optimized for analysis using RT^2^ Profiler PCR Arrays.

### Determination of Ubiquitination Gene Expression Profiles

Human Ubiquitination Pathway RT^2^ Profiler PCR Array kit (Qiagen, LLC-USA, Germantown, MD, United States) was used to measure the transcript expression levels of 84 key ubiquitination process genes that regulated degradation of cellular proteins by the UPS. In addition, the array kit incorporated five housekeeping genes controls, three reverse transcription controls, three positive PCR controls, and a human genomic DNA contamination control. Given the limited sample material for analysis from anemic children, duplicates were measured for a subset of patients (*n* = 8) and inter-assay variability was assessed. In addition, duplicate analysis was carried out for RNA isolated from malaria naïve US donors. For both the malaria samples and those from healthy US donors, intra-assay coefficient of variation (CV %) was ≤4.24. As such, individual patient RNA samples were utilized for the clinical phenotypes presented here. For each patient sample, reaction master mixes (equivalent to 96 reactions) were prepared as per manufacturers’ protocol to contain, as final concentrations, 1× RT^2^ SYBR Green mastermix and 0.5 µg cDNA synthesis mix in RNase-free water. Aliquots of 25 µl assay mix were dispensed into each well of the RT^2^ Profiler PCR Array plate (Qiagen, LLC-USA, Germantown, MD, United States). Each well contained a specified gene primer pair, mixed with an inert dye for quality control. Amplification was performed on the StepOne Plus Real-Time PCR system (Thermo Fisher Scientific, Waltham, MA, United States), at an initial denaturation temperature of 95°C for 10 min, followed by 40 cycles of 95°C for 15 s and 60°C for 1 min. To test the specificity of the amplification, dissociation curve analysis was performed, with the ramp set from 60 to 95°C.

### Data Analysis

Comparisons of categories variable was computed using Fisher’s exact test with exact *p*-values for homogeneity. Clinical and laboratory characteristics between SMA and M*l*MA groups were determined using Mann-Whitney-U test while group means were compared by a two-sided Student’s *t*-test. Multiple test correction was performed using the Bonferroni-Holm method (familywise error rate 0.050).

The cycle threshold (C_T_) value for each well were calculated using the Real-Time Cycler software (Thermo Fisher Scientific, Waltham, MA, United States). Baseline levels were defined by selecting the automated baseline option on the StepOne Plus Real Time PCR thermocycler (Thermo Fisher Scientific, Waltham, MA, United States). Threshold was set manually by using the log view of the amplification plots, and data containing the C_T_ values was exported to an Excel spreadsheet. Data was uploaded and analyzed using the RT^2^ Profiler PCR Array Data Analysis Webportal (Qiagen, LLC-USA, Germantown, MD, United States). Gene expression levels were determined using the 2^(^–^Delta Delta C_T_) method, where Fold change (calculated by converting the *ΔΔC*_*T*_ from a log_2_ scale to a linear scale; *2* ^*−ΔΔC_T_*^ was determined for each gene on the ubiquitination process (Livak and Schmittgen, 2001). Values >1 indicate upregulation of gene expression, while values between 0 and 1 denotes downregulated gene expression. To accurately infer biological relevance, fold regulation (the negative inverse of the fold change [−1/x]) values are represented. The *p*-values were calculated using the Student’s two sample *t*-test (two-sided assuming equal variances) on the *2* ^*−ΔΔC_T_*^ values for each gene to compare the SMA group with the M*l*MA group. The tests were performed at a 5% significance level (*p*-value ≤ 0.050 was considered statistically significant).

### Process Analysis of Ubiquitination Gene Expression Data

Process analysis of the genes that were differentially expressed was performed using the web-based algorithm, MetaCore™ software suite (Clarivate Analytics, Philadelphia, PA, United States). Possible networks were created according to the list of the differentially expressed genes using the MetaCore™ database (Honda et al., 2006). MetaCore™ maps the differentially expressed genes onto biological functions and canonical pathways, and identifies pathways in which significant differentially expressed genes are overrepresented. We conducted enrichment analysis of ubiquitination gene sets by comparing the SMA (case) and M*l*MA (control) groups across two functional ontologies: namely the canonical pathway maps and the gene ontology (GO) processes. Direct interactions among the genes that were differentially expressed were examined and each connection represents physical interactions experimentally confirmed (Honda et al., 2006).

## Results

### Demographic and Clinical Characteristics of the Study Participants

The primary goal of the current study was to determine if genes involved in ubiquitination process are differentially expressed among children with varying severities of malarial anemia. As such, children in the overall cohort who had HIV-1, bacteremia, cerebral malaria, sickle-cell disease, and any other identified infections were excluded from the current study. We selected children with *P. falciparum* infections and stratified individuals into two groups based on hemoglobin concentrations: Hb ≥9.0 g/dl (mild malarial anemia, M*l*MA, *n* = 23) and Hb <6.0 g/dl (severe malarial anemia, SMA, *n* = 21). The demographic, clinical and laboratory characteristics of study participants are presented in Table 1. Although the proportion of males was higher in the M*l*MA group, and *vice versa* in the SMA group, the overall gender distribution was not significantly different between the two groups (*p* = 0.069). The two groups had comparable age (*p* = 0.681) and glucose levels (*p* = 0.184). Children with SMA had a lower temperature upon admission (*p* < 0.001). Consistent with the *a priori* classification of the clinical groups according to Hb concentrations, children with SMA had decreased hematocrit (*p* < 0.001) and RBCs (*p* < 0.001). The SMA group also had an elevated RDW (*p* = 0.004), PDW (*p* = 0.016), WBC count (*p* = 0.086), and lymphocyte count (*p* = 0.022), while the neutrophil count was lower (*p* = 0.050). However, after correction for multiple testing (Holm correction) only admission temperature, hematocrit, RBCs, and RDW remained significantly different. Other hematological parameters, along with parasite densities (*p* = 0.250), were comparable between the groups (Table 1).

**TABLE 1:** Demographic, clinical, and laboratory characteristics of the study participants.

Characteristics	Total	M*l*MA (Hb ≥9.0 g/dl)	SMA (Hb <6.0 g/dl)	*p*-value
No. of participants (*n* = 44)	44	23	21	
Sex, *n* (%)				
Male	22 (50.0)	15 (65.25)	7 (33.3)	0.069^a^
Female	22 (50.0)	8 (34.8)	14 (66.7)	
Age, months	22.4 (20.81)	25.0 (21.4)	19.7 (18.0)	0.681^b^
Glucose, mmol/L	5.4 (3.1)	5.2 (3.5)	5.8 (3.2)	0.184^b^
Admission temperature, °C	37.0 (1.5)	37.9 (1.9)	37.5 (1.6)	**<0.001** ^b^
**Hematological Parameters**				
Hemoglobin, g/dl	9.4 (5.8)	10.4 (1.4)	4.8 (1.6)	NA
Hematocrit, %	28.9 (18.6)	33.9 (4.0)	15.2 (4.5)	**<0.001** ^b^
Red blood cells, × 10^6^/µl	3.9 (2.5)	4.7 (0.8)	2.2 (0.8)	**<0.001** ^b^
Red cell distribution width	18.7 (3.5)	17.7 (2.8)	20.1 (5.4)	**0.004** ^b^
Mean corpuscular volume, fL	71.9 (9.2)	70.2 (11.1)	73.0 (9.3)	0.503^b^
Mean corpuscular hemoglobin, pg	22.1 (4.2)	21.8 (4.5)	22.6 (3.7)	0.953^b^
Mean corpuscular hemoglobin concentration, g/L	31.3 (2.2)	31.8 (2.2)	31.1 (2.9)	0.165^b^
Platelets, ×10^3^/µl	118.0 (158.2)	104.0 (156.0)	125.0 (162.0)	0.869^b^
Mean platelet volume, fL	8.1 (2.3)	7.9 (2.5)	8.4 (1.8)	0.716^b^
Platelet distribution width	16.8 (1.7)	16.2 (1.3)	17.4 (1.2)	0.016^b^
White blood cell, ×10^3^/µl	12.2 (8.0)	11.7 (5.3)	15.5 (12.4)	0.086^b^
Lymphocytes, ×10^3^/µl	4.2 (3.8)	4.0 (2.2)	5.1 (7.2)	0.022^b^
Monocytes, ×10^3^/µl	0.9 (1.1)	0.9 (0.8)	1.2 (1.2)	0.352^b^
Neutrophils, ×10^3^/µl	5.5 (3.2)	6.8 (1.2)	4.0 (1.0)	0.050^b^
Granulocytes, ×10^3^/µl	7.2 (6.7)	6.9 (4.7)	9.3 (7.7)	0.520^b^
**Parasitological Indices**				
Parasite density, MPS/µl	5,497 (11,498)	3,481 (5,324)	7,705 (15,598)	0.250^c^

Data are the median (interquartile range; IQR) unless otherwise noted. Children (*n* = 44) presenting with malaria at SCRH were recruited. Based on hemoglobin (Hb) levels, children were categorized into either mild malarial anemia (M*l*MA; Hb ≥9.0 g/dl, *n* = 23) or severe malarial anemia (SMA; Hb <6.0 g/dl, *n* = 21).

a Fisher’s exact test with exact *p*-values for homogeneity was performed.

b The Mann-Whitney-U test was used to compare the M*l*MA and SMA groups.

c Group means were compared by a two-sided two Student’s *t*-test. Significant *p*-values after multiple test correction using the Bonferroni-Holm method (familywise error rate 0.050) are shown in bold. Abbreviations: MPS-malaria parasites presented as mean (standard deviation).

### Differential Expression of Ubiquitination Genes in Children With Acute Malaria

**FIGURE 1:**
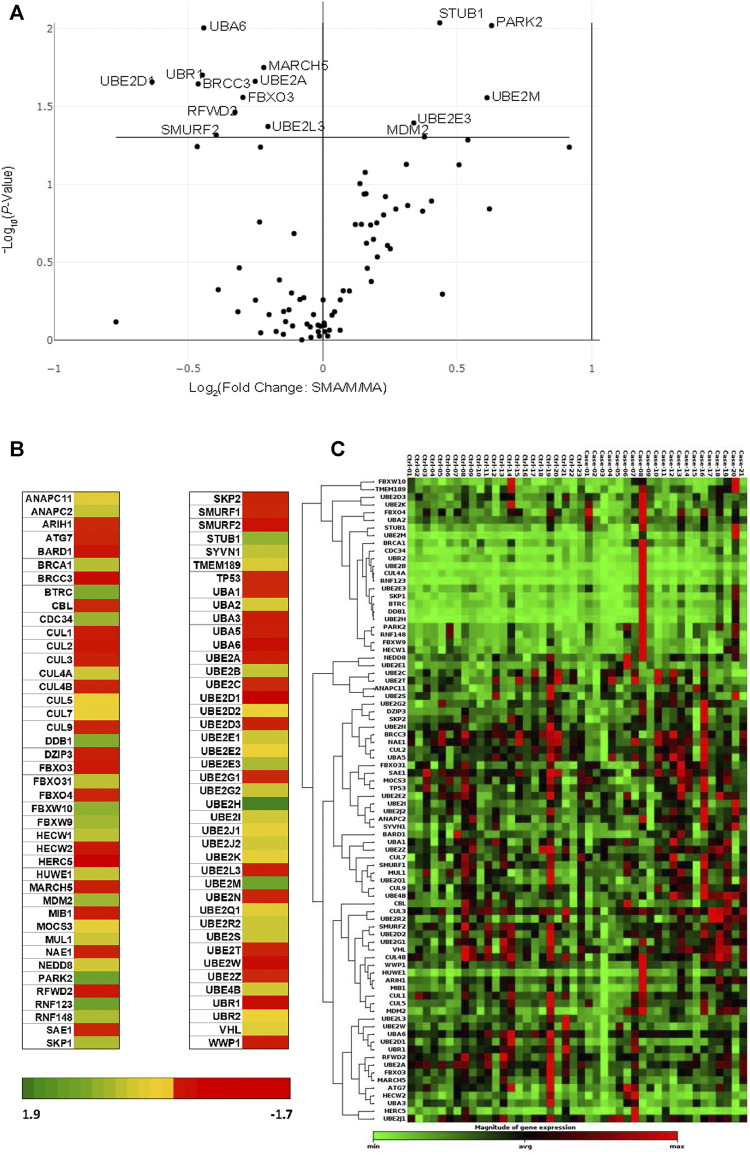
Comparison of ubiquitination gene expression levels. Children (*n* = 44), with mild malarial anemia (M*l*MA; Hb ≥9.0 g/dl, *n* = 23) and severe malarial anemia (SMA; Hb <6.0 g/dl, *n* = 21) were enrolled into the study. Gene expression profiles were measured using the Human Ubiquitylation Pathway RT^2^ Profiler PCR Array kit. Geometric mean was used as a normalization factor, and data standardized using five housekeeping genes [Actin, beta (ACTB), Beta-2-microglobulin (B2M), Glyceraldehyde-3-phosphate dehydrogenase (GAPDH), Hypoxanthine phosphoribosyltransferase 1 (HPRT1) and Ribosomal protein, large, P0 (RPLPO)]. Data were analyzed by the ΔΔ^*C_T_*^ method (2^−ΔΔ*C_T_*^) (Livak and Schmittgen, 2001), using the RT^2^ Profiler PCR Array Data Analysis Webportal (Qiagen, United States). Fold regulation set at 1.5, and *p* ≤ 0.050. **(A)**. The Volcano Plot shows gene expression changes that plots the log base 2 of each gene fold change value on the *x*-axis versus the negative log base 10 of each genes *p*-value on the *y*-axis. The center vertical line indicates unchanged gene expression, while the two outer vertical lines indicate the selected fold regulation threshold, with the data points right of the solid line indicating upregulated genes and those to the left representing downregulated genes. *p*-values were calculated using the student’s *t*-test of the triplicate raw C_T_ values. **(B)**. Heat map showing the graphical and color-coded representation of fold regulation data between M*l*MA and SMA groups overlaid onto the PCR array plate layout. The yellow color represents the average magnitude of gene expression. The brightest red represents the smallest value, and the brightest green represents the highest value. **(C)**. Cluster gram of non-supervised hierarchical clustering of the entire dataset showing a heat map with dendrograms indicating co-regulated genes across the clinical groups. The black color represents the average magnitude of gene expression. The brightest green represents the smallest value, and the brightest red represents the highest value. Similarities of genes across the PCR array was calculated using a correlation coefficient between 2 dimensional profiles.

Transcriptional profiles of 84 ubiquitination genes were measured in whole-blood from 44 children with acute malaria: M*l*MA (Hb ≥9.0 g/dl; *n* = 23) and SMA (Hb <6.0 g/dl; *n* = 21). There were 15 genes that showed significant differential regulation between the clinical groups: 10 genes were down-regulated in children with SMA (*BRCC3*, *FBXO3*, *MARCH5*, *RFWD2*, *SMURF2*, *UBA6*, *UBE2A*, *UBE2D1*, *UBE2L3*, and *UBR1*), while five genes displayed up-regulated transcript expression (*MDM2*, *PARK2*, *STUB1*, *UBE2E3*, and *UBE2M*) (Figure 1 and Supplementary Table S1). The fold-regulation for differential gene expression between the clinical groups (referenced to M*l*MA) ranged from 1.9 to −1.7 (Figure 1B). A non-supervised hierarchical cluster analysis with dendrograms was also generated to examine co-regulated genes at the individual patient level in children with M*l*MA and SMA (Figure 1C). One primary cluster of significantly altered gene expression emerged that was represented by *MDM2, UBE2L3*, *UBA6*, *UBE2D1*, *UBR1*, *RFWD2*, *UBE2A*, *FBXO 3*, and *MARCH5* with *SMURF2* being more distally related (Figure 1C).

### Gene Set Analysis in Children With Acute Malaria

**FIGURE 2:**
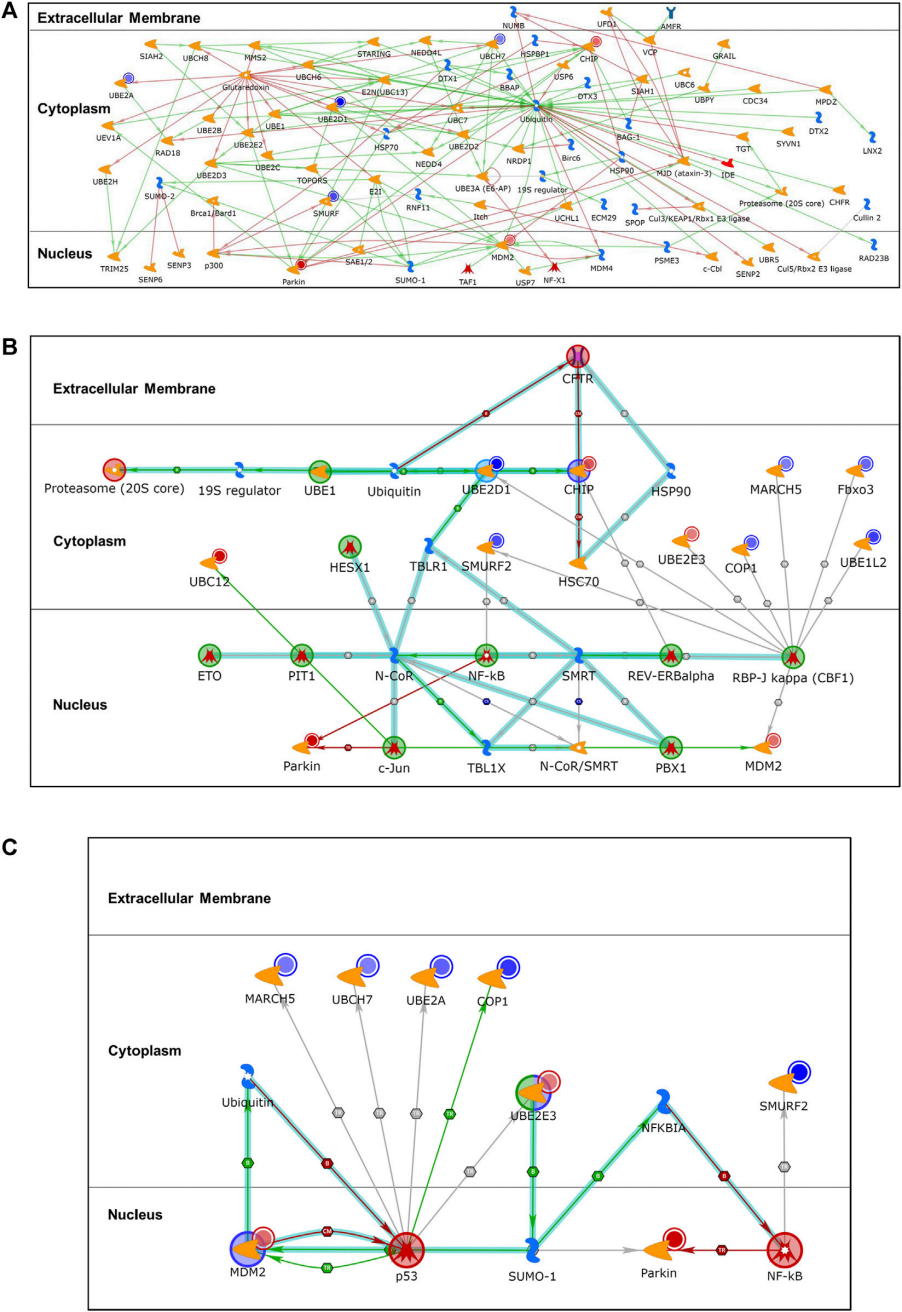
Differentially expressed gene enrichment analysis of the top scored process networks. Relationship between differentially expressed ubiquitination genes (*p* ≤ 0.050) in the case (SMA; *n* = 21) and control (M*l*MA; *n* = 23) groups was determined using enrichment analysis to identify process networks on MetaCore™. Additional enrichment analysis for same differentially expressed genes (*p* ≤ 0.050) was done using canonical pathway modeling to map out associated subnetwork processes. **(A)**. Ubiquitin-Proteasomal Proteolysis (FDR, *p* = 7.049 × 10^−11^) process network that encompassed 8 of the 15 genes that were significantly dysregulated. The blue-shaded circles show down-regulated genes and the red-shaded circles are up-regulated genes, all from the ubiquitination panel. **(B)**. The sub-network [CFTR, Proteasome (20S core), CHIP, RBP-J kappa (CBF1), c-Jun, *p* = 1.610 × 10^−41^] contains 11 seed nodes (genes with *p* < 0.050 for differential expression between SMA and M*l*MA) and 31 total nodes. **(C)**. The sub-network (p53, NF-kB, UBE2E3, MDM2, SUMO-1, *p* = 1.460 × 10^−32^) contains 8 seed nodes and 13 total nodes.

To explore the relationship among the differentially expressed ubiquitination genes (*p* ≤ 0.050), enrichment analysis for process networks was performed using MetaCore™. This analysis identified the Ubiquitin-Proteasomal Proteolysis as the top disrupted process network (FDR, *p* = 7.049 × 10^−11^) that impacted on 8 [*UBE2A*, *UBE2D1*, *SMURF1* and *SMURF2* (represented as *SMURF*), *UBE2L3* (*UBCH7*), *STUB1* (*CHIP*), *MDM2* and *PARKIN*] of the 15 differentially expressed genes (Figure 2A). Additional enrichment analyses were performed using canonical pathway modeling for the genes that were differentially expressed (*p* ≤ 0.050) between the case (SMA) and control (M*l*MA) groups. Three significant sub-networks emerged (Table 2). The top ranked sub-network [CFTR, Proteasome (20S core), CHIP, RBP-J kappa (CBF1), and c-Jun, *p* = 1.610 × 10^−41^] contained 11 significant differentially expressed seed nodes and 31 total nodes, with gene ontology (GO) processes identified for protein catabolic processes (Figure 2B and Table 2). The second-ranked sub-network (p53, NF-kB, UBE2E3, MDM2, and SUMO-1, *p* = 1.460 × 10^−32^) contained eight seed nodes and 13 total nodes with GO processes centered on protein conjugation or removal, ubiquitination, and proteolysis (Figure 2C and Table 2). The third sub-network [Synphilin 1, Alpha-synuclein, Septin 5 (CDC-REL1), GPR37, and MJD (ataxin-3), *p* = 2.20 × 10^−7^] contained only two seed nodes and 13 total nodes with GO processes involving metabolic processes for dopamine, catecholamine, catechol-containing compounds, and cellular biogenic amines (Table 2).

**TABLE 2:** Sub-networks of the pathway gene enrichment analysis for differentially expressed genes between SMA and M*l*MA groups.

Sub-network	Gene ontology processes	Total	Seed	z Score	g Score	*p*-value
CFTR, Proteasome (20S core), CHIP, RBP-J kappa (CBF1), c-Jun	Proteasomal protein catabolic process (45.2%; **6.97 × 10** ^**−17**^)	31	11	267.04	267.04	**1.61 × 10** ^**−41**^
	Protein catabolic process (54.8%; **9.62 × 10** ^**−17**^)					
	Proteolysis involved in cellular protein catabolic process (51.6%; **1.94 × 10** ^**−16**^)					
	Cellular protein catabolic process (51.6%; **4.53 × 10** ^**−16**^)					
	Ubiquitin-dependent protein catabolic process (48.4%; **1.13 × 10** ^**−15**^)					
p53, NF-kB, UBE2E3, MDM2, SUMO-1	Protein modification by small protein conjugation or removal (92.3%; **5.68 × 10** ^**−15**^)	13	8	299.92	299.92	**1.46 × 10** ^**−32**^
	Protein modification by small protein conjugation (76.9%; **4.11 × 10** ^**−12**^)					
	Protein polyubiquitination (61.5%; **7.15 × 10** ^**−12**^)					
	Protein ubiquitination (69.2%; **1.11 × 10** ^**−10**^)					
	Proteolysis (76.9%; **8.52 × 10** ^**−10**^)					
Synphilin 1, Alpha-synuclein, Septin 5 (CDC-REL1), GPR37, MJD (ataxin-3)	Dopamine metabolic process (30.8%; **1.26 × 10** ^**−08**^)	13	2	74.96	74.96	**2.20 × 10** ^**−07**^
	Catechol-containing compound metabolic process (30.8%; **5.58 × 10** ^**−08**^)					
	Catecholamine metabolic process (30.8%; **5.57 × 10** ^**−08**^)					
	Cellular biogenic amine metabolic process (30.8%; **6.63 × 10** ^**−07**^)					
	Cellular amine metabolic process (30.8%; **6.83 × 10** ^**−07**^)					

Enrichment analyses were performed by canonical pathway modeling for the differentially expressed genes (*p* < 0.050) between the case (SMA) and control (M*l*MA) groups using MetaCore™. Top-ranked gene ontology (GO) processes associated with each subnetwork are shown. Significant *p*-values are shown in bold.

## Discussion

Clinical immunity to malaria is mediated by acquisition of natural immunity following repeated episodes of infections (Ong’echa et al., 2011; Perkins et al., 2013; Ryg-Cornejo et al., 2016; Healer et al., 2018). In immune-naïve children living under intense malaria transmission, innate immunity serves as the first line of defense prior to the development of natural immunity (Ong’echa et al., 2011; Ryg-Cornejo et al., 2016). We have previously shown that variability in immune response genes influences the pathogenesis of SMA (Perkins et al., 2013). Although the molecular basis of severe malaria has not been fully elucidated, our studies have demonstrated that susceptibility to varying malaria clinical outcomes is conditioned, at least in part, by variation in genes that code for soluble mediators of inflammation (Perkins et al., 2013; Achieng et al., 2019; Anyona et al., 2020). To expand the knowledge base on the etiology of severe malaria, we investigated transcriptional profiles in a panel of 84 genes involved in the ubiquitination process in children with acute malaria.

Although ubiquitination is central for a plethora of physiological processes, which include innate and adaptive immunity, cell survival and differentiation (Popovic et al., 2014), there is lack of knowledge whether dysregulation of host gene expression for ubiquitination impacts the pathogenesis of SMA. Here, we identified 15 ubiquitination genes that were differentially expressed in children with mild versus severe malarial anemia. Enrichment analyses revealed that these genes are represented within a single process network: Ubiquitin-Proteasomal Proteolysis. Additional analyses using GO enrichment to determine how the differentially expressed genes relate to biological processes, cellular components, and molecular functions (Ekins et al., 2007) identified sub-networks involved in proteasomal protein and protein catabolic processes, as well as ubiquitin-dependent catabolic processes.

The cohort was stratified into discrete (polarized) phenotypes [M*l*MA (Hb ≥9.0 g/dl, *n* = 23] and SMA (Hb <6.0 g/dl, *n* = 21)] to enrich for potential signals and create non-overlapping categories with regards to clinical manifestations. Since malaria-infected children in this region are often co-infected with HIV-1 and/or bacteremia, and these pathogens promote more severe disease manifestations (Kendall et al., 1980; Aluoch, 1997; Aidoo et al., 2002; Otieno et al., 2006; Kifude et al., 2007; Davenport et al., 2010; Waitumbi et al., 2010; Were et al., 2011), children with co-infections were not included in the current study. Although currently unknown, it is also possible that endemic co-infections in children with malaria may impact on ubiquitination. In addition, children who received prior medication were excluded from the study.

Of the 84 ubiquitination genes explored, 15 were differentially expressed in children with SMA compared to M*l*MA. Although previously unexplored as part of the host immune response to clinical malaria, these novel results highlight the importance of the ubiquitination process in the pathogenesis of malarial anemia. Enrichment network analysis of genes that were differentially expressed in SMA versus M*l*MA identified the Ubiquitin-Proteasomal Proteolysis process as the top-ranked network (Figure 2A), suggesting enhanced targeting of proteins for degradation by the ubiquitin-proteosome pathway in children with complicated *P. falciparum* infections. This finding is further reinforced by the GO analyses which identified cellular activities encompassing catabolic processing of proteasomal and cellular proteins through hydrolysis of peptide bonds mediated by the proteasome (Saric et al., 2004) as the highest ranked sub-network: CFTR, Proteasome (20S core), CHIP, RBP-J kappa (CBF1), and c-Jun (Figure 2B). Consistent with these cellular processes forming the highest ranked sub-network, the non-supervised hierarchical cluster analysis revealed one primary cluster of co-expressed genes that encompassed most of the differentially expressed genes within this sub-network.

Central to the ubiquitin-proteasomal proteolysis process is the relative balance between activation and inhibition of ubiquitin following binding of various signaling proteins. Ubiquitin has a major role in targeting cellular proteins for degradation by the 26S proteosome (Pickart, 1997). Degradation of proteins via the proteasome involves an initial polyubiquitination of substrate proteins targeted for elimination through the ubiquitination enzyme cascade involving the ubiquitin activation (E1), conjugation (E2), and protein substrate labeling (E3) enzymes (Ciechanover, 2005). Ubiquitin is transferred from E1 to the catalytic cysteine residue of the E2 active site and subsequently to E3 (Pickart and Eddins, 2004). The ability of E3 enzyme to target specific proteins for ubiquitination suggests a mechanism that enables selective removal of specific proteins, allowing for precise regulation of cellular functions (Ardley and Robinson, 2005; Berndsen and Wolberger, 2014). However, this selectivity is subject to efficient transfer of ubiquitin to the cysteine residue of E2 active site. Cysteines undergo oxidative and reduction modifications post-transcriptionally to allow signaling and protein processing (Matsui et al., 2020). One of the steps involves cysteine oxidation to form sulfenic acid, followed by two subsequent reduction reactions, namely formation of S-glutathionylated protein from reduced glutathione, and a subsequent reduction through a chemical or enzymatic process to remove the modification (Matsui et al., 2020). Under physiological circumstances, these processes permit oxidation-reduction signaling and shields cysteines from irreversible oxidation (Matsui et al., 2020). Results from our network analysis revealed glutaredoxin as a central enzyme in the overall process for inhibition of signaling proteins through covalent modifications (Figure 2A). Targets of glutaredoxin [i.e., *UBE2A*, *UBE2D1*, and *UBCH7* (*UBE2L3*)] were downregulated in children with SMA, suggesting that severe disease is defined by an impairment in antioxidant defense.

In addition, our investigations revealed that mRNAs for the nuclear proteins, parkin and MDM2, were upregulated in children with SMA. *PARK2* encodes the parkin protein, which acts as a cytosolic ubiquitin-E3- ligase whose main function is to regulate mitophagy (Wang et al., 2015). As such, upregulation of parkin in children with SMA suggests enhanced mitochondrial damage due to cellular stress. MDM2 is a negative regulator of the p53 tumor suppressor, and functions as an E3 ubiquitin ligase responsible for the ubiquitination and degradation of p53, thereby repressing p53 transcriptional activity (Haupt et al., 1997; Kubbutat et al., 1997). Previous studies in children with *P. falciparum* malaria (6–11 years of age) showed that elevated monocytic expression of p53 attenuates the inflammatory process and is associated with favorable clinical outcomes (Tran et al., 2019). Thus, upregulation of MDM2 in the context of reduced p53 in children with SMA could be an important mechanism for increased pathogenesis (Figure 1B). Consistent with this hypothesis, the MDM2-p53 interaction appears to play a central role in the downregulation of *MARCH5*, *UBE2L3* (*UBCH7*), *UBE2A*, and *RFWD2* (*COP1*) that emerged from the GO sub-network analyses (i.e., p53, NF-kB, UBE2E3, MDM2, and SUMO-1; Figure 2C). This sub-network is important for protein conjugation, removal, ubiquitination, polyubiquitination, and proteolysis. In addition, the GO sub-network analyses identified metabolic processing of dopamine, catecholamines, and cellular biogenic amines: [Synphilin 1, Alpha-synuclein, Septin 5 (CDC-REL1), GPR37, MJD (ataxin-3)]. Since catecholamines stimulate erythrocytic signaling pathways that result in altered red blood cell properties (e.g., cell flexibility, deformability, and filterability), these results appear to suggest increased metabolic derangements in children with SMA (Oonishi et al., 1997; Tuvia et al., 1998; Tuvia et al., 1999).

Collectively, utilization of a targeted panel of 84 human ubiquitination genes identified differentially expressed mRNA transcripts between children with SMA versus M*l*MA, indicating for the first time, the importance of the host ubiquitination process in the pathogenesis of SMA. These findings are consistent with the central role of ubiquitination in cellular processes for cancer, infections, muscle dystrophies, autoimmunity, inflammatory disorders, metabolic syndromes, and neurodegenerative diseases [reviewed in (Popovic et al., 2014)]. Limitations of the current study include targeting of 84 genes amongst a much broader number of signaling proteins involved in the ubiquitination process. The reduced number of genes likely limited our ability to detect additionally important signaling pathways and processes with the enrichment analyses. Studies are ongoing in our laboratory to validate our current findings in a larger cohort [during acute infection (day 0) and at post-treatment, well visit (day 14)] using a multi-omics approach (genomics, transcriptomics, and proteomics) to discover additionally important ubiquitination genes involved in the pathogenesis of severe malaria.

## Data Availability

The original contributions presented in the study are included in the article/Supplementary Material, further inquiries can be directed to the corresponding author.
