# Supplementary material for: Differential Gene Expression in Host Ubiquitination Processes in Childhood Malarial Anemia
Source: Front Genet. 2021 Nov 22;12:764759. doi: 10.3389/fgene.2021.764759 (PMC8646022; doi:10.3389/fgene.2021.764759)
Supplement: Supplementary file 1 [file Table1.docx]

**SUPPLEMENTARY TABLE 1**

**Differential expression of ubiquitylation genes between mild malarial anemia (M*l*MA) and severe malarial anemia (SMA)**

| **Genes** | | | | **2^(^−^Avg. (Delta(Ct))** | | **Fold Change** | **Fold Regulation** | ***P*-value** |
| --- | --- | --- | --- | --- | --- | --- | --- | --- |
| **Refseq** | **Symbol** | **Description** | **Gname** | **M*l*MA Group** | **SMA Group** | **SMA Group** | |  |
| NM_001002244 | ANAPC11 | Anaphase promoting complex subunit 11 | APC11/ Apc11p/ HSPC214 | 0.028592 | 0.029899 | 1.05 | 1.05 | 0.552 |
| NM_013366 | ANAPC2 | Anaphase promoting complex subunit 2 | APC2 | 0.002641 | 0.003033 | 1.15 | 1.15 | 0.177 |
| NM_005744 | ARIH1 | Ariadne homolog, ubiquitin-conjugating enzyme E2 binding protein, 1 (Drosophila) | ARI/HARI/ HHARI/ UBCH7BP | 0.019744 | 0.019274 | 0.98 | -1.02 | 0.686 |
| NM_006395 | ATG7 | ATG7 autophagy related 7 homolog (*S. cerevisiae*) | APG7-LIKE/ APG7L/ GSA7 | 0.016909 | 0.015939 | 0.94 | -1.06 | 0.549 |
| NM_000465 | BARD1 | BRCA1 associated RING domain 1 | - | 0.001934 | 0.001478 | 0.76 | -1.31 | 0.475 |
| NM_007294 | BRCA1 | Breast cancer 1, early onset | BRCAI/ BRCC1/ BROVCA1/ FANCS/ IRIS/ PNCA4/ PPP1R53/ PSCP/ RNF53 | 0.003639 | 0.004389 | 1.21 | 1.21 | 0.144 |
| NM_024332 | BRCC3 | BRCA1/BRCA2-containing complex, subunit 3 | BRCC36/ C6.1A/ CXorf53 | 0.002670 | 0.001938 | 0.73 | -1.38 | **0.023** |
| NM_033637 | BTRC | Beta-transducin repeat containing | BETA-TRCP/ FBW1A/ FBXW1/ FBXW1A/ FWD1/ bTrCP/ bTrCP1/ betaTrCP | 0.005316 | 0.007731 | 1.45 | 1.45 | 0.052 |
| NM_005188 | CBL | Cas-Br-M (murine) ecotropic retroviral transforming sequence | C-CBL/ CBL2/ FRA11B/ NSLL/ RNF55 | 0.014790 | 0.014343 | 0.97 | -1.03 | 0.960 |
| NM_004359 | CDC34 | Cell division cycle 34 homolog (S. cerevisiae) | E2-CDC34/ UBC3/ UBCH3/ UBE2R1 | 0.012604 | 0.016662 | 1.32 | 1.32 | 0.128 |
| NM_003592 | CUL1 | Cullin 1 | - | 0.018444 | 0.015674 | 0.85 | -1.18 | 0.175 |
| NM_003591 | CUL2 | Cullin 2 | - | 0.010868 | 0.008767 | 0.81 | -1.24 | 0.344 |
| NM_003590 | CUL3 | Cullin 3 | CUL-3/ PHA2E | 0.027425 | 0.024778 | 0.90 | -1.11 | 0.918 |
| NM_003589 | CUL4A | Cullin 4A | - | 0.018757 | 0.020979 | 1.12 | 1.12 | 0.239 |
| NM_003588 | CUL4B | Cullin 4B | CUL-4B/ MRXHF2/ MRXS15/ MRXSC/ SFM2 | 0.014632 | 0.013498 | 0.92 | -1.08 | 0.499 |
| NM_003478 | CUL5 | Cullin 5 | VACM-1/ VACM1 | 0.015206 | 0.015253 | 1.00 | 1.00 | 0.777 |
| NM_014780 | CUL7 | Cullin 7 | 3M1/ KIAA0076/ dJ20C7.5 | 0.000829 | 0.000839 | 1.01 | 1.01 | 0.942 |
| NM_015089 | CUL9 | Cullin 9 | H7AP1/ PARC | 0.000612 | 0.000566 | 0.93 | -1.08 | 0.812 |
| NM_001923 | DDB1 | Damage-specific DNA binding protein 1, 127kDa | DDBA/ UV-DDB1/ XAP1/ XPCE/ XPE/ XPE-BF | 0.045528 | 0.064732 | 1.42 | 1.42 | 0.075 |
| NM_014648 | DZIP3 | DAZ interacting protein 3, zinc finger | PPP1R66/ UURF2/ hRUL138 | 0.003592 | 0.003128 | 0.87 | -1.15 | 0.686 |
| NM_012175 | FBXO3 | F-box protein 3 | FBA/ FBX3 | 0.007330 | 0.005968 | 0.81 | -1.23 | **0.028** |
| NM_024735 | FBXO31 | F-box protein 31 | FBX14/ FBXO14/ Fbx31/ MRT45/ pp2386 | 0.001933 | 0.002282 | 1.18 | 1.18 | 0.247 |
| NM_012176 | FBXO4 | F-box protein 4 | FBX4 | 0.003652 | 0.003460 | 0.95 | -1.06 | 0.997 |
| NM_031456 | FBXW10 | F-box and WD repeat domain containing 10 | Fbw10/ HREP/ SM25H2/ SM2SH2 | 0.000106 | 0.000145 | 1.36 | 1.36 | 0.507 |
| NM_032301 | FBXW9 | F-box and WD repeat domain containing 9 | Fbw9 | 0.001381 | 0.001785 | 1.29 | 1.29 | 0.149 |
| NM_015052 | HECW1 | HECT, C2 and WW domain containing E3 ubiquitin protein ligase 1 | NEDL1 | 0.000293 | 0.000348 | 1.19 | 1.19 | 0.259 |
| NM_020760 | HECW2 | HECT, C2 and WW domain containing E3 ubiquitin protein ligase 2 | NEDL2 | 0.002257 | 0.001814 | 0.80 | -1.24 | 0.658 |
| NM_016323 | HERC5 | Hect domain and RLD 5 | CEB1/CEBP1 | 0.021497 | 0.012605 | 0.59 | -1.71 | 0.765 |
| NM_031407 | HUWE1 | HECT, UBA and WWE domain containing 1 | ARF-BP1/ HECTH9/ HSPC272/ Ib772/ LASU1/ MULE/ URE-B1/ UREB1 | 0.009895 | 0.011566 | 1.17 | 1.17 | 0.157 |
| NM_017824 | MARCH5 | Membrane-associated ring finger (C3HC4) 5 | MARCH-V/ MITOL/ RNF153 | 0.018974 | 0.016300 | 0.86 | -1.16 | **0.018** |
| NM_002392 | MDM2 | Mdm2 p53 binding protein homolog (mouse) | ACTFS/ HDMX/ hdm2 | 0.040180 | 0.052151 | 1.30 | 1.30 | **0.050** |
| NM_020774 | MIB1 | Mindbomb homolog 1 (Drosophila) | DIP-1/DIP1/ LVNC7/ MIB/ ZZANK2/ ZZZ6 | 0.004968 | 0.004178 | 0.84 | -1.19 | 0.554 |
| NM_014484 | MOCS3 | Molybdenum cofactor synthesis 3 | UBA4 | 0.006070 | 0.006255 | 1.03 | 1.03 | 0.658 |
| NM_024544 | MUL1 | Mitochondrial E3 ubiquitin protein ligase 1 | C1orf166/ GIDE/ MAPL/ MULAN/ RNF218 | 0.006585 | 0.007380 | 1.12 | 1.12 | 0.346 |
| NM_003905 | NAE1 | NEDD8 activating enzyme E1 subunit 1 | A-116A10.1/ APPBP1/ HPP1/ ula-1 | 0.009833 | 0.008794 | 0.89 | -1.12 | 0.411 |
| NM_006156 | NEDD8 | Neural precursor cell expressed, developmentally down-regulated 8 | NEDD-8 | 0.086901 | 0.095569 | 1.10 | 1.10 | 0.099 |
| NM_004562 | PARK2 | Parkinson protein 2, E3 ubiquitin protein ligase (parkin) | AR-JP/ LPRS2/ PDJ/ PRKN | 0.000026 | 0.000040 | 1.55 | 1.55 | **0.010** |
| NM_022457 | RFWD2 | Ring finger and WD repeat domain 2 | COP1/ RNF200 | 0.035864 | 0.028615 | 0.80 | -1.25 | **0.034** |
| NM_022064 | RNF123 | Ring finger protein 123 | FP1477/ KPC1 | 0.015263 | 0.023464 | 1.54 | 1.54 | 0.144 |
| NM_198085 | RNF148 | Ring finger protein 148 | - | 0.000026 | 0.000032 | 1.24 | 1.24 | 0.075 |
| NM_005500 | SAE1 | SUMO1 activating enzyme subunit 1 | AOS1/ HSPC140/ SUA1/ UBLE1A | 0.020912 | 0.019910 | 0.95 | -1.05 | 0.535 |
| NM_006930 | SKP1 | S-phase kinase-associated protein 1 | EMC19/ OCP-II/ OCP2/ SKP1A/ TCEB1L/ p19A | 0.004607 | 0.005729 | 1.24 | 1.24 | 0.137 |
| NM_005983 | SKP2 | S-phase kinase-associated protein 2 (p45) | FBL1/ FBXL1/ FLB1/ p45 | 0.002746 | 0.002517 | 0.92 | -1.09 | 0.639 |
| NM_020429 | SMURF1 | SMAD specific E3 ubiquitin protein ligase 1 | - | 0.004832 | 0.004638 | 0.96 | -1.04 | 0.788 |
| NM_022739 | SMURF2 | SMAD specific E3 ubiquitin protein ligase 2 | - | 0.004721 | 0.003589 | 0.76 | -1.32 | **0.048** |
| NM_005861 | STUB1 | STIP1 homology and U-box containing protein 1, E3 ubiquitin protein ligase | CHIP/ HSPABP2/ NY-CO-7/ SCAR16/ SDCCAG7/ UBOX1 | 0.028766 | 0.038833 | 1.35 | 1.35 | **0.009** |
| NM_172230 | SYVN1 | Synovial apoptosis inhibitor 1, synoviolin | DER3/ HRD1 | 0.005692 | 0.006684 | 1.17 | 1.17 | 0.120 |
| NM_199129 | TMEM189 | Transmembrane protein 189 | KUA | 0.000728 | 0.000779 | 1.07 | 1.07 | 0.484 |
| NM_000546 | TP53 | Tumor protein p53 | BCC7/ LFS1/ P53/ TRP53 | 0.031665 | 0.031293 | 0.99 | -1.01 | 0.882 |
| NM_003334 | UBA1 | Ubiquitin-like modifier activating enzyme 1 | A1S9/ A1S9T/ A1ST/ AMCX1/ CFAP124/ GXP1/ POC20/ SMAX2 / UBA1A/ UBE1/ UBE1X | 0.070721 | 0.070084 | 0.99 | -1.01 | 0.943 |
| NM_005499 | UBA2 | Ubiquitin-like modifier activating enzyme 2 | ARX/ HRIHFB2115/ SAE2 | 0.036345 | 0.039509 | 1.09 | 1.09 | 0.181 |
| NM_003968 | UBA3 | Ubiquitin-like modifier activating enzyme 3 | NAE2/ UBE1C/ hUBA3 | 0.035758 | 0.030461 | 0.85 | -1.17 | 0.058 |
| NM_198329 | UBA5 | Ubiquitin-like modifier activating enzyme 5 | THIFP1/ UBE1DC1 | 0.004461 | 0.003956 | 0.89 | -1.13 | 0.880 |
| NM_018227 | UBA6 | Ubiquitin-like modifier activating enzyme 6 | E1-L2/ MOP-4/ UBE1L2 | 0.023495 | 0.017300 | 0.74 | -1.36 | **0.010** |
| NM_003336 | UBE2A | Ubiquitin-conjugating enzyme E2A | HHR6A/ MRXS30/ MRXSN/ RAD6A/ UBC2 | 0.045637 | 0.038354 | 0.84 | -1.19 | **0.022** |
| NM_003337 | UBE2B | Ubiquitin-conjugating enzyme E2B | E2-17kDa/ HHR6B/ HR6B/ RAD6B/ UBC2 | 0.223072 | 0.256568 | 1.15 | 1.15 | 0.293 |
| NM_181803 | UBE2C | Ubiquitin-conjugating enzyme E2C | UBCH10/ dJ447F3.2 | 0.003971 | 0.003947 | 0.99 | -1.01 | 0.814 |
| NM_003338 | UBE2D1 | Ubiquitin-conjugating enzyme E2D 1 | E2(17)KB1/ SFT/ UBC4/5/ UBCH5/ UBCH5A | 0.014066 | 0.009068 | 0.64 | -1.55 | **0.022** |
| NM_181838 | UBE2D2 | Ubiquitin-conjugating enzyme E2D 2 | E2(17)KB2/ PUBC1/ UBC4/ UBC4/5/ UBCH4/ UBCH5B | 0.003232 | 0.003245 | 1.00 | 1.00 | 0.805 |
| NM_181893 | UBE2D3 | Ubiquitin-conjugating enzyme E2D 3 | E2(17)KB3/ UBC4/5/ UBCH5C | 0.209877 | 0.189750 | 0.90 | -1.11 | 0.655 |
| NM_182666 | UBE2E1 | Ubiquitin-conjugating enzyme E2E 1 | UBCH6 | 0.042862 | 0.047882 | 1.12 | 1.12 | 0.115 |
| NM_152653 | UBE2E2 | Ubiquitin-conjugating enzyme E2E 2 | UBCH8 | 0.003498 | 0.003515 | 1.00 | 1.00 | 0.882 |
| NM_006357 | UBE2E3 | Ubiquitin-conjugating enzyme E2E 3 | UBCH9/ UbcM2 | 0.022757 | 0.028743 | 1.26 | 1.26 | **0.040** |
| NM_003342 | UBE2G1 | Ubiquitin-conjugating enzyme E2G 1 | E217K/ UBC7/ UBE2G | 0.006477 | 0.006271 | 0.97 | -1.03 | 0.823 |
| NM_182688 | UBE2G2 | Ubiquitin-conjugating enzyme E2G 2 | UBC7 | 0.004216 | 0.004801 | 1.14 | 1.14 | 0.225 |
| NM_182697 | UBE2H | Ubiquitin-conjugating enzyme E2H | E2-20K/ GID3/ UBC8/ UBCH/ UBCH2 | 0.090882 | 0.171544 | 1.89 | 1.89 | 0.058 |
| NM_003345 | UBE2I | Ubiquitin-conjugating enzyme E2I | C358B7.1/ P18/ UBC9 | 0.061892 | 0.068996 | 1.11 | 1.11 | 0.084 |
| NM_016021 | UBE2J1 | Ubiquitin-conjugating enzyme E2, J1, U | CGI-76/ HSPC153/ HSPC205/ HSU93243/ NCUBE-1/ NCUBE1/ UBC6/ UBC6E/ Ubc6p | 0.056426 | 0.057369 | 1.02 | 1.02 | 0.864 |
| NM_194458 | UBE2J2 | Ubiquitin-conjugating enzyme E2, J2 | NCUBE-2/ NCUBE2/ PRO2121 | 0.014038 | 0.015607 | 1.11 | 1.11 | 0.116 |
| NM_005339 | UBE2K | Ubiquitin-conjugating enzyme E2K | E2-25K/ HIP2/ HYPG/ LIG/ UBC1 | 0.056180 | 0.057518 | 1.02 | 1.02 | 0.692 |
| NM_003347 | UBE2L3 | Ubiquitin-conjugating enzyme E2L 3 | E2-F1/ L-UBC/ UBCH7/ UbcM4 | 0.050855 | 0.044160 | 0.87 | -1.15 | **0.043** |
| NM_003969 | UBE2M | Ubiquitin-conjugating enzyme E2M | UBC-RS2/ UBC12/ hUbc12 | 0.013756 | 0.021018 | 1.53 | 1.53 | **0.028** |
| NM_003348 | UBE2N | Ubiquitin-conjugating enzyme E2N | HEL-S-71/ UBC13/ UBCHBEN; UBC13/ UbcH-ben/ UbcH13 | 0.039631 | 0.036797 | 0.93 | -1.08 | 0.207 |
| NM_017582 | UBE2Q1 | Ubiquitin-conjugating enzyme E2Q family member 1 | GTAP/ NICE-5/ PRO3094/ UBE2Q | 0.026754 | 0.028188 | 1.05 | 1.05 | 0.482 |
| NM_017811 | UBE2R2 | Ubiquitin-conjugating enzyme E2R 2 | CDC34B/ E2-CDC34B/ UBC3B | 0.033683 | 0.038077 | 1.13 | 1.13 | 0.183 |
| NM_014501 | UBE2S | Ubiquitin-conjugating enzyme E2S | E2-EPF/ E2EPF/ EPF5 | 0.002300 | 0.002604 | 1.13 | 1.13 | 0.421 |
| NM_014176 | UBE2T | Ubiquitin-conjugating enzyme E2T (putative) | FANCT/ HSPC150/ PIG50 | 0.006371 | 0.005789 | 0.91 | -1.10 | 0.761 |
| NM_018299 | UBE2W | Ubiquitin-conjugating enzyme E2W (putative) | UBC-16/ UBC16 | 0.036013 | 0.026076 | 0.72 | -1.38 | 0.057 |
| NM_023079 | UBE2Z | Ubiquitin-conjugating enzyme E2Z | HOYS7/ USE1 | 0.018944 | 0.018709 | 0.99 | -1.01 | 0.802 |
| NM_006048 | UBE4B | Ubiquitination factor E4B | E4/ HDNB1/ UBOX3/ UFD2/ UFD2A | 0.003041 | 0.003357 | 1.10 | 1.10 | 0.181 |
| NM_174916 | UBR1 | Ubiquitin protein ligase E3 component n-recognin 1 | JBS | 0.007852 | 0.005762 | 0.73 | -1.36 | **0.020** |
| NM_015255 | UBR2 | Ubiquitin protein ligase E3 component n-recognin 2 | C6orf133/ bA49A4.1/ dJ242G1.1/ dJ392M17.3 | 0.044471 | 0.044500 | 1.00 | 1.00 | 0.552 |
| NM_000551 | VHL | Von Hippel-Lindau tumor suppressor | HRCA1/ RCA1/ VHL1/ pVHL | 0.029839 | 0.031192 | 1.05 | 1.05 | 0.864 |
| NM_007013 | WWP1 | WW domain containing E3 ubiquitin protein ligase 1 | AIP5/Tiul1/ hSDRP1 | 0.007501 | 0.006394 | 0.85 | -1.17 | 0.897 |
| NM_001101 | ACTB | Actin, beta | BRWS1/ PS1TP5BP1 | 7.928675 | 6.826271 | 0.86 | -1.16 | 0.070 |
| NM_004048 | B2M | Beta-2-microglobulin | - | 9.944494 | 8.253799 | 0.83 | -1.20 | 0.091 |
| NM_002046 | GAPDH | Glyceraldehyde-3-phosphate dehydrogenase | G3PD/ GAPD/ HEL-S-162eP | 0.518613 | 0.609274 | 1.17 | 1.17 | 0.082 |
| NM_000194 | HPRT1 | Hypoxanthine phosphoribosyltransferase 1 | HGPRT/ HPRT | 0.015887 | 0.016328 | 1.03 | 1.03 | 0.665 |
| NM_001002 | RPLP0 | Ribosomal protein, large, P0 | L10E/ LP0/ P0 /PRLP0/ RPP0 | 1.539355 | 1.784114 | 1.16 | 1.16 | 0.174 |

Data presented as fold regulation of differentially expressed genes (n=84) of the ubiquitylation pathway. Children (n=44), with mild malarial anemia (M*l*MA; Hb≥9.0g/dl, n=23) and severe malarial anemia (SMA; Hb<6.0g/dl, n=21) were enrolled into the study. Gene expression profiles were measured using the Human Ubiquitylation Pathway RT² Profiler PCR Array kit. Fold-Change (2^−Δ^*^C^_T_*) represents the normalized gene expression (2^−Δ^*^C^_T_*) in the SMA group divided the normalized gene expression (2^−Δ^*^C^_T_*) in the M*l*MA group. The data were normalized with 5 housekeeping genes [Actin, beta (ACTB), Beta-2-microglobulin (B2M), Glyceraldehyde-3-phosphate dehydrogenase (GAPDH), Hypoxanthine phosphoribosyltransferase 1 (HPRT1) and Ribosomal protein, large, P0 (RPLPO)]. Data were analyzed by the ΔΔ*C_T_* method (2^−ΔΔ^*^C^_T_*), using the RT^2^ Profiler PCR Array Data Analysis Webportal (Qiagen, USA). *p*-values were calculated using the student’s *t*-test of the raw C_T_ values. *p-*value set at ≤0.050 and those significant are shown in bold. Three genes with a fold change ≥1.5, also represented as a fold regulation to infer biological significance. The gene encoding the Ubiquitin-conjugating enzyme E2D 1 (UBE2D1; fold regulation = -1.55, *p* = 0.022) expression was down-regulated in children with SMA. Genes encoding the Parkinson protein 2, E3 ubiquitin protein ligase (PARK2; fold regulation = 1.55, *p* = 0.010) and Ubiquitin-conjugating enzyme E2M (UBE2M; fold regulation = 1.53, *p* = 0.028) were upregulated in children with SMA relative to M*l*MA.
